# FDG PET/CT response in diffuse large B-cell lymphoma: Reader variability and association with clinical outcome

**DOI:** 10.1097/MD.0000000000004983

**Published:** 2016-09-30

**Authors:** Eun Ji Han, Joo Hyun O, Hyukjin Yoon, Seung Eun Jung, Gyeongsin Park, Byung Ock Choi, Seok-Goo Cho

**Affiliations:** a Department of Radiology, Daejeon St. Mary's Hospital, College of Medicine, The Catholic University of Korea, Daejeon; b Department of Radiology; c Department of Pathology; d Department of Radiation Oncology; e Department of Hematology, Seoul St. Mary's Hospital, College of Medicine, The Catholic University of Korea, Seoul, Republic of Korea.

**Keywords:** DLBCL, FDG PET/CT, outcome, reader variability, response criteria

## Abstract

F-18-fluoro-2-deoxyglucose (FDG) positron emission tomography/computed tomography (PET/CT) is essential for monitoring response to treatment in patients with diffuse large B-cell lymphoma (DLBCL) and qualitative interpretation is commonly applied in clinical practice. We aimed to evaluate the interobserver agreements of qualitative PET/CT response in patients with DLBCL and the predictive value of PET/CT results for clinical outcome.

PET/CT images were obtained for patients with DLBCL 3 times: at baseline, after 3 cycles of first-line chemotherapy (interim), and after completion of chemotherapy. Two nuclear medicine physicians (with 3 and 8 years of experience with PET/CT) retrospectively assessed response to chemotherapy blinded to the clinical outcome using International Harmonization Project (IHP) criteria and Deauville 5-point score. The associations between PET/CT results and progression-free survival (PFS) and overall survival (OS) were assessed using Cox regression analysis.

A total of 112 PET/CT images were included from 59 patients with DLBCL (36 male, 23 female; mean age 53 ± 14 years). Using the IHP criteria, interobserver agreement was substantial (Cohen κ = 0.76) with absolute agreement consistency of 89%. Using the Deauville score, interobserver agreement was moderate (Cohen weighted κ = 0.54) and absolute consistency was 62%. The most common cause of disagreements was discordant interpretation of residual tumor uptake. With median follow-up period of 60 months, estimated 5-year PFS and OS were 81% and 92%, respectively. Neither interim nor posttreatment PET/CT results by both readers were significantly associated with PFS. Interim PET/CT result by the more experienced reader using Deauville score was a significant factor for OS (*P* = 0.019).

Moderate-to-substantial interobserver agreement was observed for response assessments according to qualitative PET/CT criteria, and interim PET/CT result could predict OS in patients with DLBCL. Further studies are necessary to further standardize the PET/CT-based response criteria for more consistent interpretation.

## 1 Introduction

F-18-fluoro-2-deoxyglucose (FDG) positron emission tomography (PET) and PET/computed tomography (CT) are used worldwide for evaluation and management of various malignancies.^[1]^ Because PET/CT has high diagnostic accuracy, especially for aggressive types of lymphoma compared to conventional imaging studies such as CT or magnetic resonance imaging, and has predictive value for outcomes,^[2,3]^ it is recommended as an essential staging tool of various Hodgkin and non-Hodgkin lymphomas including diffuse large B-cell lymphoma (DLBCL). In addition, PET/CT is applied for assessment of response to first-line chemotherapy and subsequent treatment planning in DLBCL.^[4,5]^ Recent reports showed interim PET/CT, performed midway through the chemotherapy, to be useful for predicting treatment response and prognosis.^[6]^ Thus, risk-adapted and response-adapted therapies based on interim PET/CT results have been actively investigated.^[7,8]^

Accurate response assessment directs the decision for either treatment continuance or regimen change and can provide predictive value for prognosis.^[9]^ Various criteria are currently used to assess treatment response by imaging studies in the clinical setting, and the most widely used Response Evaluation Criteria in Solid Tumors and World Health Organization criteria are based on anatomical, more specifically, size changes.^[10–12]^ In malignant lymphoma, residual masses are frequently observed even after successful treatment because of fibrosis or necrosis. Residual masses do not always represent viable tumors and do not indicate poorer outcomes.^[13,14]^ Therefore, FDG uptake-based approach is applied for evaluation of metabolic change in lymphoma.^[9]^ FDG PET/CT images can be evaluated qualitatively or quantitatively, and the qualitative analysis is more commonly applied in response assessment of lymphoma. The current National Comprehensive Cancer Network (NCCN) guidelines recommended that FDG PET images should be interpreted via Lugano classification incorporating the Deauville 5-point score (D5PS), which is based on visual assessment.^[5,15]^ Revised International Harmonization Project (IHP) response criteria accept simple binary decision for PET-based response.^[16]^ The degree of interobserver variation and reproducibility are important in these qualitative analyses, which have pivotal role in the patient management.

The purpose of our study was to evaluate the interobserver agreements of qualitative PET/CT response criteria in patients with DLBCL and analyze the possible causes of disagreements in response interpretation between readers. In addition, we evaluated the predictive value of PET/CT for long-term outcome.

## 2 Methods

### 2.1 Patient populations

We retrospectively reviewed 112 FDG PET/CT images of 59 consecutive patients with DLBCL taken between December 2006 and March 2012. FDG PET/CT images were performed at baseline (baseline PET/CT); after 3 cycles of rituximab, cyclophosphamide, hydroxydaunomycin, oncovin, and prednisone (R-CHOP) chemotherapy (interim PET/CT); and after completion of chemotherapy (posttreatment PET/CT). Cases with low FDG uptake in lymphoma lesions, below the hepatic activity, in the baseline PET/CT images were considered to be unassessable by FDG PET/CT and excluded from this study. Clinicopathologic variables such as age, sex, Eastern Cooperative Oncology Group performance status, Ann Arbor stage, lactate dehydrogenase (LDH) titer, extranodal involvement, bone marrow involvement, splenomegaly, international prognostic index score at diagnosis, and survival outcomes were obtained from medical and imaging records.

This study was approved by the institutional review board of Seoul St. Mary's Hospital, The Catholic University of Korea. The ethical committee of our institution waived the need for requiring patient consent for this retrospective review of imaging studies and clinical data.

### 2.2 FDG PET/CT imaging

All patients fasted for at least 6 hours before the PET/CT studies. FDG (370–555 MBq) was injected intravenously, and scanning began 60 minutes later. No patient had blood glucose levels greater than 150 mg/dL before injection. No intravenous contrast agent was administered. Studies were acquired using combined PET/CT in-line systems, Biograph Duo or Biograph Truepoint (Siemens Medical Solutions, Knoxville, TN). All patients were in a supine position. CT began at the orbitomeatal line and progressed to the upper thigh using a standard protocol: 130 kVp, 30 mA, 5-mm slice thickness (Biograph Duo); 120 kV, 50 mA, 5-mm slice thickness (Biograph Truepoint). PET followed immediately over the same body region. Acquisition time was 2 to 3 minutes per bed position. CT data were used for attenuation correction, and images were reconstructed using standard ordered-subset expectation maximization.

### 2.3 Image analysis

All PET/CT images were retrospectively assessed for response to chemotherapy by 2 nuclear medicine physicians, blinded to the clinical data and to each other's assessments. Reader A was a nuclear medicine resident with 3 years of experience in interpretation of FDG PET/CT images. Reader B was a board-certified nuclear medicine physician with 8 years of experience in interpretation of FDG PET/CT images and had periodically participated in lymphoma multidisciplinary meetings. All images were viewed at a workstation with fusion software (Syngo; Siemens) that provided multiplanar reformatted images and displayed PET images after attenuation correction, CT images, and PET/CT fusion images.

Interim or posttreatment PET/CT images were compared with the baseline PET/CT images using 2 visual analysis methods: the IHP response criteria and Lugano classification. In the IHP response criteria, last revised in 2007, the 4 recommended response categories are complete remission (CR), partial remission, stable disease, and progressive disease, and PET-based response is based on simple binary decision (PET-positive or PET-negative). PET-negative is considered to be moderately sized or large residual lesions (more than 2 cm at the greatest transverse diameter) with FDG uptake isointense or less intense than mediastinal blood pool structures. For smaller lesions less than 2 cm in diameter, FDG uptake that cannot be differentiated from surrounding background activity is considered negative because of the partial volume effect.^[16]^ The readers recorded the sites of residual lesions or newly detected lesions when they interpreted the case as PET-positive. Lugano classification for response assessment of PET/CT incorporating the D5PS is as follows: score 1, no FDG uptake above background; score 2, FDG uptake ≤ mediastinum; score 3, FDG uptake > mediastinum but ≤ liver; score 4, FDG uptake moderately > liver; and score 5, FDG uptake markedly higher than liver and/or new lesions.^[15]^ The readers recorded the sites of increased FDG uptake lesions or new lesions in cases interpreted as having score of 2, 3, 4, or 5. Based on the current NCCN guidelines, the PET/CT results by D5PS were then dichotomized using hepatic uptake as the reference (score 1, 2, or 3 vs score 4 or 5).^[5]^

### 2.4 Statistical analysis

Categorical variables were expressed as an absolute number and percentage, and continuous variables were expressed as median or mean ± standard deviation and range. Interobserver agreement was measured using McNemar test and Cohen κ. A κ value of 0.0 to 0.2 was considered to represent slight agreement; 0.21 to 0.4, fair; 0.41 to 0.6, moderate; 0.61 to 0.8, substantial; and 0.81 to 1.0, almost perfect.^[17,18]^

The primary endpoint was overall survival (OS), which was defined as the time from the date of baseline PET/CT to the date of death from DLBCL. Progression-free survival (PFS) was defined as the time from the date of baseline PET/CT to the date progression was detected, or date of last clinical follow-up. The Kaplan–Meier method and log-rank test were used to estimate the 5-year PFS and OS. A Cox proportional hazards model provided univariate analysis for the identification of significant prognostic factors for OS. Statistical analysis was carried out using SPSS software version 21.0 (IBM, Armonk, NY). A 95% confidence interval (CI) was calculated, and a *P* value less than 0.05 was considered significant.

## 3 Results

### 3.1 Patient characteristics

A total of 55 interim and 57 posttreatment PET/CT images of 59 patients with DLBCL (36 male, 23 female; mean age 53 ± 14 years) were included. At baseline, 10 patients (17%) were Ann Arbor stage I, 17 (29%) were stage II, and 32 (54%) had advanced stage. Bone marrow involvement was confirmed by biopsy in 12 patients (20%), and 26 patients (44%) had elevated LDH titers. The general characteristics of patients are in Table 1.

**Table 1:**
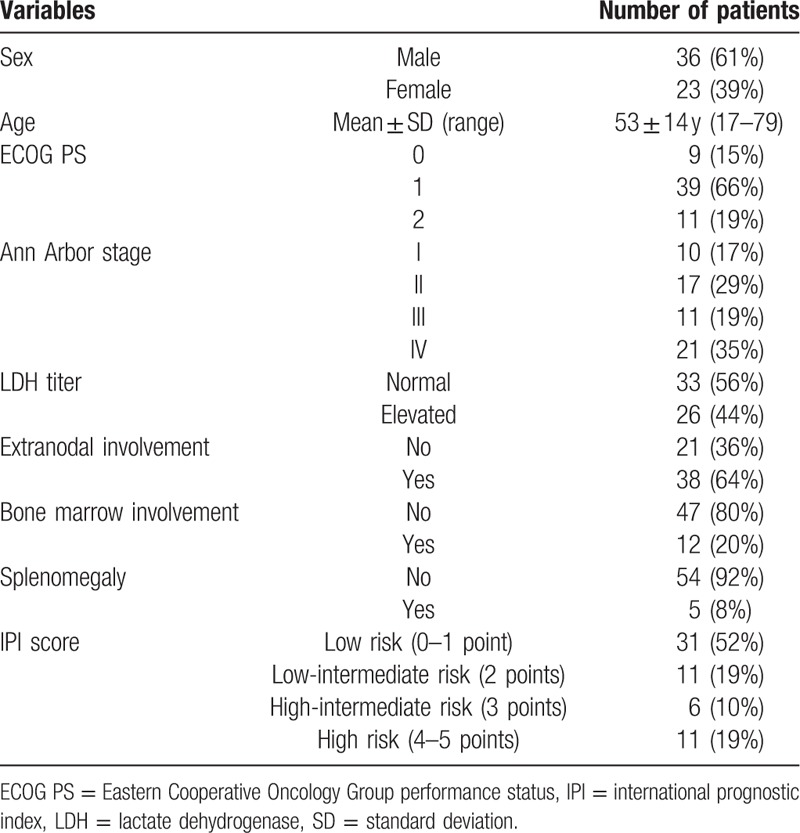
General characteristics of 59 patients.

### 3.2 Interobserver agreement of PET/CT response

Of a total of 112 PET/CT images using the IHP criteria, reader A interpreted 77 as PET-negative and 35 as PET-positive; reader B interpreted 73 as PET-negative and 39 PET-positive (Table 2). There was substantial interobserver agreement (Cohen κ = 0.76) with absolute agreement consistency of 89% (100 of 112). No significant differences were seen between readers in interpretations of interim (*P* = 0.250, McNemar test), posttreatment (*P* = 1.000), and both time point assessments (*P* = 0.388). Most of the disagreements (11 of 12) were due to inconsistencies in interpretation of residual FDG uptake. In 1 case, the disagreement was due to different interpretation of lung and pleural lesions in the posttreatment PET/CT.

**Table 2:**
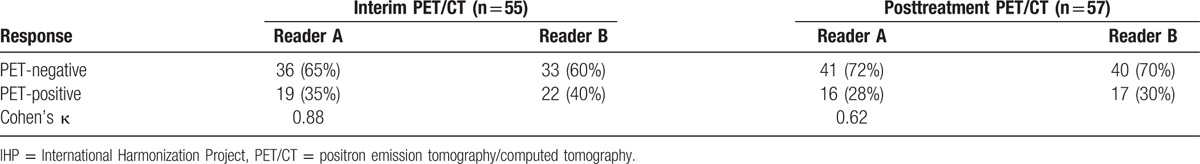
Response assessment using IHP response criteria.

The interpretation results by the 2 readers using the D5PS are given in Table 3. For a total of 112 PET/CT images, the interobserver agreement was moderate (Cohen κ = 0.43 and weighted κ = 0.54) and the absolute consistency was 62% (69 of 112). Of the 43 disagreeing cases, 1-grade disagreement was the most frequent (n = 30). The causes of disagreements were discordant interpretation of residual tumor uptake in areas with very low background activity such as cervical nodal chains, lungs, and mesentery (n = 18) or very high physiologic activity such as tonsils, bowel, and bone marrow (n = 17). In the remaining 8 disagreeing cases, there were concomitant benign conditions such as postinflammatory change, reactive lymph nodes, and postoperative change. When the results of D5PS were dichotomized as score 1, 2, or 3 versus score 4 or 5, there was 82% agreement for interim PET/CT (Cohen κ = 0.41), 88% agreement for posttreatment PET/CT (Cohen κ = 0.52), and 85% agreement for both time points (Cohen κ = 0.46). Interpretation results of a total of 112 PET/CT images showed significant difference between readers (*P* = 0.013, McNemar test).

**Table 3:**
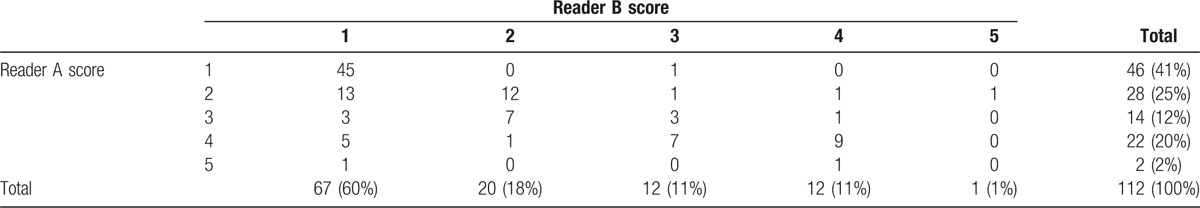
Response assessment using D5PS.

### 3.3 Survival prediction

The median follow-up period was 60 months (range 7–103): the median observation time was 60 months (range 24–103) for the surviving patients, and the median survival time was 33 months (range 7–86) for the deceased patients. Of 59 patients, 13 patients presented with tumor progression during follow-up, and the estimated 5-year PFS was 81%. The prognostic performances of PET/CT results are given in Table 4. Neither interim nor posttreatment PET/CT results were significantly associated with PFS, although the tendency was noted for the interim PET/CT response by reader B using Deauville score (*P* = 0.068). Of 50 patients with negative posttreatment PET/CT (Deauville score 1–3), 12 (24%) had progression. Of 7 patients with positive posttreatment PET/CT (Deauville score 4–5), 1 patient had progression with PFS of 64.3 months, 2 patients had additional radiotherapy following positive posttreatment PET/CT findings and are presently in ongoing CR, and 4 patients have now been in CR without additional therapy.

**Table 4:**
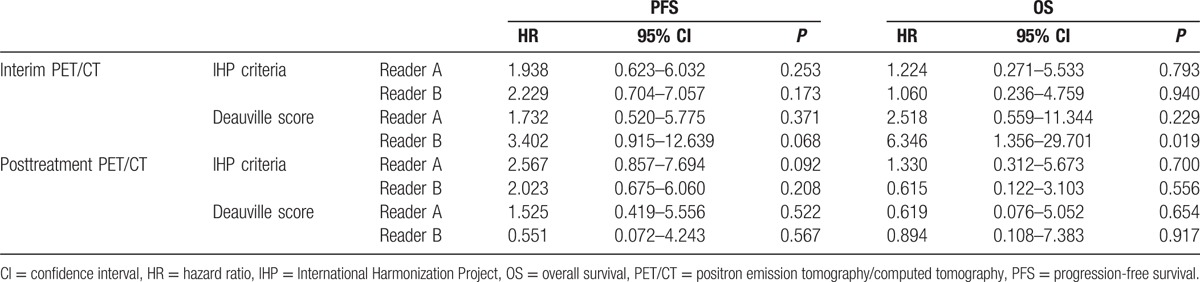
Survival prediction by PET/CT results.

**Figure 1:**
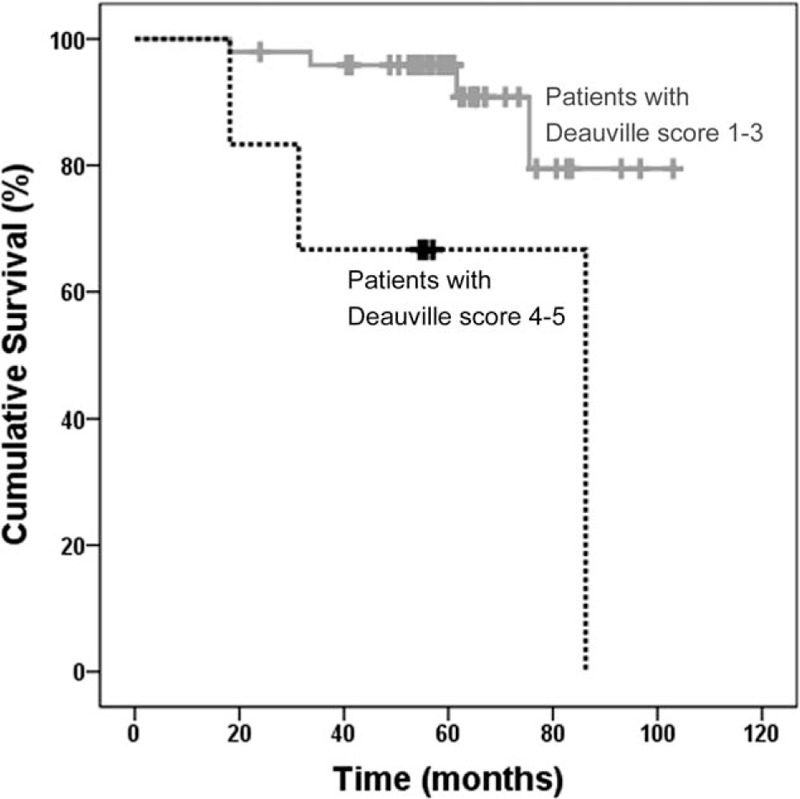
Kaplan–Meier curve of overall survival (OS). OS was significantly higher in patients with Deauville score 1, 2, or 3 than in those with score 4 or 5 in interim PET/CT images (*P* = 0.008). PET/CT = positron emission tomography/computed tomography.

During follow-up, 8 of the 59 patients died and the estimated 5-year OS was 92%. One patient's death was due to a cause unrelated to lymphoma. Forty-five patients remained alive without tumor progression. Four patients had tumor progression, but remained alive after salvage or palliative treatment. Estimating predictive power of PET/CT results by readers, interim PET/CT result by reader B using Deauville score was the only significant factor for OS (Table 4). Of 8 deceased patients, only 1 patient had positive posttreatment PET/CT with Deauville score 4 and the other 7 patients had negative posttreatment PET/CT with Deauville score 1 or 2. The mean OS in patients with Deauville score 1, 2, or 3 in interim PET/CT images (95 months, 95% CI 86.715–102.507) was significantly longer than patients with Deauville score 4 or 5 (66 months, 95% CI 37.099–94.423; *P* = 0.008, log-rank test) (Fig. 1).

## 4 Discussion

One purpose of this study was to evaluate the interobserver agreements for 2 qualitative PET/CT response criteria widely used for patients with DLBCL. Substantial interobserver agreement was seen for the IHP criteria, whereas moderate interobserver agreement was seen for D5PS. When results of D5PS were dichotomized, the interobserver variability was slightly increased (Cohen κ from 0.43 to 0.46). Previous studies using mediastinal uptake as reference demonstrated Cohen κ ranging from 0.445 to 0.65.^[19,20]^ In previous studies of response assessment using D5PS, few reported the interobserver agreement for all 5 points of the scoring method, and only a fair degree of agreement was observed with Cohen κ ranging from 0.13 to 0.387.^[21,22]^ The dichotomization of the D5PS (score 1–3 vs score 4–5) produced higher agreement with Cohen κ ranging from 0.502 to 0.96.^[19,21,23]^ Overall, the results of our study and previous studies show a wide range of interobserver agreement in qualitative PET analysis.

**Figure 2:**
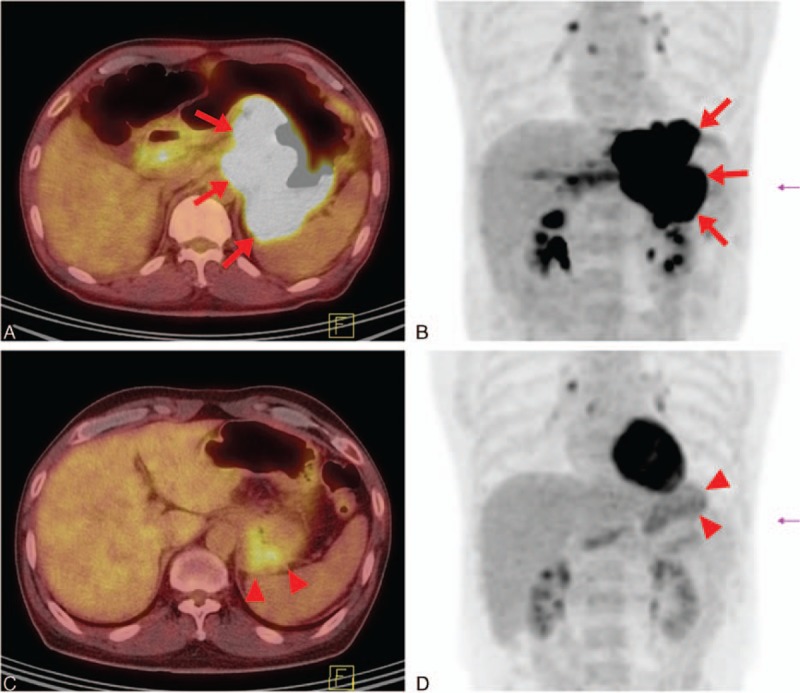
A 53-year-old man with gastric diffuse large B-cell lymphoma. In baseline positron emission tomography/computed tomography (PET/CT) transaxial (A) and PET maximum intensity projection (MIP) (B) images, intense F-18-fluoro-2-deoxyglucose (FDG) uptake was seen along the gastric wall from cardia to body, mainly along the lesser curvature side (maximum standardized uptake value 28.2) (arrows). In interim PET/CT transaxial (C) and PET MIP (D) images after 3 cycles of first-line chemotherapy, previously noted FDG uptake in the stomach showed markedly decreased intensity and extent (arrowheads). Remaining FDG uptake could not be differentiated from physiological gastric activity. Reader A interpreted this finding as Deauville score 4 and reader B interpreted it as score 1. After completion of first-line chemotherapy, the patient remains in clinical remission without additional therapy (overall survival 76.8 months). CT = computed tomography, FDG = F-18-fluoro-2-deoxyglucose, MIP = maximum intensity projection, PET = positron emission tomography.

Mikhaeel et al^[24,25]^ used the term of “minimal residual uptake (MRU)” for interim PET in patients with aggressive lymphoma. MRU is defined as residual low FDG uptake in previously involved site that is likely to represent inflammation from which small volume of residual viable tumor cannot be definitely excluded and indicates neither positive nor negative PET findings. MRU designation is subjective and depends on the reader's experience, reference point, and various elements of the imaging protocols.^[26,27]^ In our study, most of the disagreements between PET-negative and PET-positive cases were due to inconsistencies in interpreting the residual FDG uptake. Using D5PS, 1- or 2-grade disagreements were observed in 18 cases due to discordant interpretation of the presence of residual tumor uptake in areas of very low background activity such as lungs or fat tissue, and most of them were disagreements between score 1 and score 2. Similarly, observer variability study by Zijlstra et al^[28]^ reported that most common interpretation of equivocal for viable lymphoma was noted in the neck, around the abdominal aorta and iliac chains, and in the lungs. In the other end of the FDG uptake spectrum, discordant interpretations were in areas of high physiologic activity (n = 17) or concomitant benign condition (n = 8) (Fig. 2). Using the IHP criteria, 1 disagreeing case was due to discordant interpretation of increased extent of lung and pleural lesions. The lesion was from active inflammation when correlating with clinical history. Infectious or inflammatory lung lesions are frequently noted in patients with malignant lymphoma, and the IHP reported that new lung nodules are mostly benign in patients without history of pulmonary involvement.^[16,29]^ In results of our study, interim PET/CT results by the less-experienced reader (reader A) were not significantly associated with PFS and OS. In PET/CT results using visual assessment, disagreeing interpretations can occur according to readers’ training and expertise.

The second purpose of this study was to evaluate the predictive value of PET/CT results for clinical outcome. The OS was significantly different between interim PET/CT-positive and PET/CT-negative patients in our study, whereas PFS could not be predicted. Several studies reported that interim PET/CT has significant predictive value for PFS and OS in patients with DLBCL.^[30,31]^ However, a systemic review including 7 studies of 311 DLBCL patients, the prognostic value of interim PET/CT was described as having conflicting results.^[32]^ In a prospective multicenter study by Mamot et al,^[34]^ interim PET/CT after 2 cycles of R-CHOP chemotherapy was significantly associated with event-free survival, but not with OS. Heterogeneities of patient populations, therapy strategies, and the timing of the scans might explain these differences. Interim PET/CT has been performed after 1, 2, 3, or 4 cycles of chemotherapy, and no consensus is formed on the optimal timing for interim assessment. In patients with DLBCL, interim PET in modern therapeutic era including rituximab tends to have lower positive predictive value than before. Some studies reported that increased false-positive rate of interim PET is a concern with the long half-life and unique mechanisms of cytotoxicity of rituximab.^[20,33]^

For posttreatment PET/CT as well, the reports on prognostic potential have been mixed.^[6,35,36]^ Recent studies reported that PET/CT interpretation using Deauville score better predicts outcome than IHP criteria.^[37,38]^ Our results demonstrated that posttreatment PET/CT applying the Deauville score was not significantly associated with PFS and OS and showed low positive predictive value of 14%. In addition, 24% of the patients had progression despite negative posttreatment PET/CT results. Our patients had longer follow-up period with median of 5 years compared to previous studies^[35–37]^ and showed relatively longer PFS and OS with only 8 deaths during the follow-up period. Of 8 deceased patients, 1 patient with positive posttreatment PET/CT result had the longest PFS of 64.3 months and OS of 86.3 months, whereas the 7 other patients with negative posttreatment PET/CT had relatively short PFS with median of 8.3 months (range 6.4–27.8). Small number of events and potent salvage therapy options might cause failure to assign significant association between posttreatment PET/CT and OS.

Although qualitative PET analysis has great advantage of easy application and is commonly used in evaluation of lymphoma, quantitative analysis is often used for evaluation of other solid tumors.^[9,39]^ Quantitative assessment shows excellent intraobserver and interobserver reproducibility.^[40,41]^ In an international confirmatory study by Itti et al,^[19]^ interim PET/CT had higher interobserver reproducibility and prognostic value when using percentage change of maximum standardized uptake value than using Deauville score. Other previous studies reported that in DLBCL interim assessment, quantitative method has higher prognostic value compared to the visual assessment.^[30,42]^ Lugano classification suggested that Deauville score 3 should be carefully interpreted depending on the timing of assessment, the clinical context, and the treatment choice.^[15]^ Quantitative analysis could provide supplementary information to enhance the prognostic value and accuracy of PET/CT in response assessment of DLBCL.

In conclusion, an unsatisfactory interobserver agreement was observed for response assessment of PET/CT using visual analysis in patients with DLBCL who underwent first-line R-CHOP chemotherapy. Posttreatment PET/CT results using Deauville score had limited value for prediction of outcome. More standardized or detailed visual analysis method, or application of quantitative response assessment, should be considered for patients with DLBCL.
